# Supplementary material for: Are carbon emissions trading and green financial instruments synergistic? -Comprehensive quantitative research based on content analysis
Source: PLoS One. 2024 Mar 7;19(3):e0298601. doi: 10.1371/journal.pone.0298601 (PMC10919601; doi:10.1371/journal.pone.0298601)
Supplement: S1 Data — (ZIP) [file pone.0298601.s001.zip › Data/地级市Z_F工作报告.zip!/║╙▒▒/▓╫╓▌╩╨/└┤╫╘╣1⁄2╓┌║┼í╛┬φ┐╦╩2╛▌═°í┐_2018.docx]

各位代表：

现在，我代表沧州市人民政府，向大会报告工作，请予审议，并请市政协委员和列席会议的同志提出意见。

一、2017年工作回顾

刚刚过去的一年，是本届政府履职“赶考”的第一年，也是全市上下攻坚克难、砥砺奋进的一年。在省委、省政府和市委的坚强领导下，我们坚持以习近平新时代中国特色社会主义思想为指导，紧紧围绕建设创新驱动经济强市、生态宜居美丽沧州目标，解放思想、改革创新、奋发进取、乘势而上，全市经济保持稳定健康发展，社会事业全面进步。全年完成地区生产总值3816.9亿元，增长7%，经济总量稳居全省第三；一般公共预算收入240.3亿元，增长9%；固定资产投资3710.3亿元，增长6.6%；规模以上工业增加值1412.8亿元，增长5.1%；社会消费品零售总额1355.2亿元，增长10.4%；城乡居民人均可支配收入达到31044元和12363元，分别增长8.5%和9%。主要经济指标增速高于全省平均水平，规上工业增加值、固定资产投资、一般公共预算收入增速均居全省第三位。

（一）沿海开发建设成效显著。着眼发挥渤海新区龙头带动作用，大力发展港口经济，加快开发开放步伐。20万吨级航道正式通航，煤炭港区四期工程竣工验收，综合保税区具备封关运营条件；黄骅港完成吞吐量2.7亿吨、集装箱65.4万标箱，分别增长10.4%和8.7%，吞吐量位居全国26个主要港口第13位。临港产业加速聚集，中科院微电子产业园、巴安水务海水淡化、北京协和原料药等187个重点项目扎实推进，泰恒特钢40万吨铬铁合金、中储粮物流等48个项目竣工投产，恒大童世界、碧桂园生态城、沧海文化风景区开工建设，成为新区绿色发展新地标。渤海新区成立10年来，地区生产总值增长2.5倍、达到567亿元，一般公共预算收入增长7.6倍、超过50亿元，分别占全市的1/6和1/5，成为拉动全市经济发展的强力引擎。

（二）协同发展迈出坚实步伐。全方位对接京津、承接转移，全市京津产业合作项目1100多个，总投资5524亿元。北京现代最新车型ix35正式上市，北汽威旺黄骅基地新能源汽车实现量产；北京生物医药产业园入驻药企102家，27家开工建设、8家竣工投产；华北石化千万吨炼油完成整体工程的80%，成品油全部达到国六标准；明珠国际服饰产业特色小镇，是京津冀协同发展产业转移投资和体量最大的轻工生产项目，一期正式开工，签约企业4000余家。与天津滨海新区、东丽区、红桥区签订战略合作协议，与首都师范大学展开深入合作，北师大任丘附属学校、北京忠德学校肃宁分校建成招生，解放军301医院国家老年病研究中心沧州合作医院揭牌成立。京津冀协同发展沧州剧场，奏响新的乐章。

（三）产业转型升级亮点纷呈。坚持以“六个一”工程为抓手，推进工业提档升级，全年实施亿元以上技改项目467个；新增规上工业企业201家，总数达到2428家。战略性新兴产业提速发展，华为沧州云计算中心正式上线，运河区激光产业园被中国光学会授予“北方光谷”称号；全市汽车及相关产业实现增加值122.5亿元，增长21%。科技型中小企业、省级以上科技创新服务平台分别增加1300家和27家；万人发明专利拥有量1.26件、增长61.5%，增速全省第二。质量品牌建设扎实推进，中国驰名商标达到32件，沧海核装备PDCA管理经验被评为“全省工业企业质量标杆”，肃宁、东光被国家质检总局分别命名为全国裘皮服装、包装机械产业知名品牌创建示范区。全市新增规上服务业企业108家，第三产业对GDP增长贡献率达到66.5%；成功举办首届旅发大会，旅游综合收入突破163亿元，增长31.8%。现代农业稳步发展，全市新增省级现代农业园区6个，土地流转率37.3%，新型农业经营主体超1.4万家，温氏200万头生猪、中捷犇放牧业5万头奶牛等重点项目竣工投产，农业产业化率达到65%。全市推进转型发展的信心更加坚定，步履更加坚实。

（四）城乡面貌发生明显变化。围绕提升品位、增强辐射带动力，持续加大中心城区建设投入。沧州动物园建成开园，九河路全线贯通，青少年宫、妇女儿童活动中心如期竣工；水月寺、清真寺等重点片区26条道路完成雨污分流改造；西部城区调峰锅炉建成投用，市区新增集中供热面积580万平方米；启动“一区三边”治理行动，拆除违法建筑251万平方米。县城建设全面升级，黄骅、东光被评为国家园林城市（县城）；盐山县盐山镇、沧县兴济镇等8个镇入围“2017年全国综合实力千强镇”；青县马厂镇、东光县连镇等12个镇列入省重点培育的100个特色小城镇名单，数量全省第二。美丽乡村建设全面推进，启动27个“三区同建”示范区项目，594个省级重点村完成改造提升任务。全市常住人口城镇化率达到52.6%。以城带乡、城乡一体、统筹发展的格局初步形成。

（五）改革开放全面扎实推进。持续深化“放管服”改革，取消下放市级审批事项377项，市本级行政审批时限削减55.3%，行政审批局实现县域全覆盖。深入推进“多证合一”“证照联办”，新增市场主体8.1万户，总数达到38万户。开发区四项改革进展顺利，聘任制全面推行，任丘开发区列入“河北京南国家科技成果转移转化示范区”，临港、高新区等5个省级开发区主营业务收入超过500亿元。16家国有集体危困企业退出，市级机关内设机构改革和精简人员编制工作完成。新增上市企业19家，总数达到148家，均居全省第二位；农信社改革稳步推进，市金融控股公司组建运营。成功举办公共服务项目洽谈周、第五届海外侨商沧州行、燕赵之光海归博士基层创新创业行等系列招商推介活动。全市实际利用外资6.24亿美元、外贸进出口247亿元，分别增长10.4%和31.7%。改革激发的动力、开放带来的活力正在逐步释放。

（六）生态环境质量持续改善。启动实施清洁能源供暖、燃煤锅炉淘汰、散乱污企业整治等六大攻坚行动，推广气（电）代煤14.6万户，淘汰燃煤锅炉7569台，整治散乱污企业6628家，中心城区实行鞭炮禁售禁放，全市空气质量综合指数、PM2.5平均浓度分别下降4%和4.3%。大气污染热点网格化管理试点工作实现精准监控、靶向治理，得到环保部充分肯定。圆满完成地下水超采综合治理三年试点工作，深层地下水水位止降回升；城市集中式饮用水源地水质达标率稳定保持100%。黄骅盐碱地生态修复项目获“中国人居环境范例奖”。启动大运河沿线和中心城区高速公路围合区造林增绿行动计划，全市完成造林面积53万亩，森林覆盖率达到30.2%。生态环境全民共治、精准施治的理念深入人心，行动更加自觉。

（七）脱贫攻坚取得重要进展。用“绣花功夫”全力打好脱贫攻坚战，海兴、南皮成功摘帽出列，全市244个贫困村、4.48万贫困人口实现脱贫退出。扎实开展贫困人口建档立卡“回头看”，清退6.32万人、新纳入1.61万人。深入实施“五个一批”工程，全年投入各类扶贫资金51.6亿元。突出产业扶贫，建成村级光伏电站184个，1.3万贫困户受益。强化政策兜底，在全省率先推出“3+2健康扶贫模式”，从源头上阻断因病因灾致贫返贫问题，被国家作为“海兴案例”在全国推广。

（八）各项社会事业全面进步。全市财政用于民生支出443.6亿元，增长8.9%，年初承诺的十件民生实事全部完成。多渠道拓宽就业，城镇新增就业9.37万人。沧县、南皮等5个县（市）达到国家义务教育发展基本均衡县评估认定标准，沧州师院顺利通过教育部高等院校本科教学工作合格评估。大力推进医联体建设和分级诊疗试点工作，京津冀医保跨省异地就医实现直接结算。全市棚户区改造新开工1.16万套、改造农村危房2035户，超额完成省定任务。引黄入冀补淀和李家岸引黄线路正式通水，南水北调受水区全部完成水源切换，260万人饮用长江水。运动休闲城市建设全面启动，国家公共文化服务体系示范区创建工作稳步实施，成功举办第十六届中国吴桥国际杂技艺术节沧州会场、第十四届全国武术之乡武术比赛等系列文体活动。有力有效推进军队退役人员安置工作，国防后备力量建设基础更加巩固。安全生产形势总体平稳，平安沧州建设扎实推进。民族宗教、外事侨务、人民防空、气象地震、史志档案、妇女儿童、老龄、残疾人等事业都取得了新进步。

各位代表，一年来，我们以迎接党的十九大胜利召开和学习贯彻十九大精神为动力，旗帜鲜明讲政治，聚精会神抓发展，铭责于心、扛责在肩，多点发力、精准施策，坚定自觉地推动党中央和省、市重大决策部署落地生根、落实见效。面对省委、省政府的信任和嘱托，我们坚持率先发展总要求，全力打造亮点、事事争创一流，石四药搬迁、北航科技园、传化智能公路港等重大战略支撑项目取得突破性进展，规上工业企业总数、外贸进出口增速等多项指标全省第一，热点网格、健康扶贫、公立医院改革等典型经验做法走在全国前列；面对集中凸显的矛盾和问题，我们坚持照单全收理旧账，不推脱、不回避，迎难而上、因势利导，成功啃下危困企业改制、散乱污企业治理、违法建筑拆除等“硬骨头”，一批与群众利益息息相关的历史遗留问题得到有效解决；面对各种风险和挑战，我们坚持沉着应对守底线，依法处置滨海大宗问题，妥善做好信访维稳工作，为党的十九大胜利召开创造了良好环境；面对人民群众的期待和企盼，我们坚持担当实干讲奉献，深入开展“两学一做”专题教育，大力转作风、优环境、走新路、抓落实，政府自身建设不断加强，施政能力和服务水平不断提升，全年办理人大代表建议205件、政协提案574件，按时办复率100%。

各位代表，看似寻常最奇崛，成如容易却艰辛。过去一年取得的成绩，是省委、省政府和市委正确领导的结果，归功于760万沧州儿女的团结拼搏，离不开市人大、市政协的监督支持。在此，我代表沧州市人民政府，向全市人民，向人大代表、政协委员，向各民主党派、工商联、人民团体和各界人士，向驻沧解放军、武警官兵和政法干警，向所有关心支持沧州发展的同志们、朋友们，致以崇高的敬意和衷心的感谢！

在看到成绩的同时，我们也清醒地认识到我市发展不平衡不充分的问题依然突出。主要是：发展质量和效益不高，“稳”的基础还不牢固，新旧动能转换不快；科技创新能力弱，研发投入少，实体经济水平有待提升；生态环境治理任重道远，实现持续好转仍需付出艰苦努力；民生改善还有薄弱环节，社会建设管理、公共服务水平还不高；政府职能转变还需加快，作风建设仍需加强，公务人员干事创业能力与新形势、新任务要求还有差距。对这些矛盾和问题，我们一定高度重视，下大力加以解决。

二、2018年目标任务和重点工作

中国特色社会主义进入新时代，沧州也站上新的历史起点，进入了全新的发展阶段。这是一个历史性窗口期和战略性机遇期叠加凸显的阶段，也是一个产业融合竞进、新旧动能接续转换的阶段，更是一个为建设创新驱动经济强市、生态宜居美丽沧州奠定坚实基础的阶段。推进沧州新一轮发展，我们有习近平新时代中国特色社会主义思想的强大政治引领，有省委、省政府和市委的正确领导，有稳定向好的外部条件，有京津冀协同发展、雄安新区规划建设、“一带一路”等重大战略机遇，有港口、区位、交通、土地等综合优势，有全市干部群众人心思进、人心思干、凝心聚力、奋发进取的强大正能量。我们一定要准确把握沧州未来发展的阶段性特征，审时度势、精心谋划，切实按照市委九届三次全会提出的坚持“六个统一”和展现“八个新作为”要求，紧紧抓住用好机遇，有效应对困难挑战，集中智慧、集中力量把沧州的事情办好，努力在新时代展现新气象、创造新业绩。

2018年，是贯彻党的十九大精神的开局之年，是改革开放40周年，是决胜全面建成小康社会、实施“十三五”规划承上启下的关键一年。今年政府工作的总体要求是：全面贯彻党的十九大精神，坚持以习近平新时代中国特色社会主义思想为指导，坚持稳中求进工作总基调，坚持新发展理念，紧扣我国社会主要矛盾变化，按照高质量发展的要求，统筹推进“五位一体”总体布局和协调推进“四个全面”战略布局，以供给侧结构性改革为主线，推动质量变革、效率变革、动力变革，聚焦创新驱动和转型升级、京津冀协同发展、港口经济、乡村振兴、深化改革、治理污染、新型城镇化、改善民生和创新社会治理等重点工作，促进经济社会高质量发展，打造沿海率先发展创新示范区、建设环渤海地区重要工业城市，奋力开创新时代建设创新驱动经济强市、生态宜居美丽沧州新局面。

经济社会发展的主要目标是：地区生产总值增长7%以上，一般公共预算收入增长7.5%，固定资产投资增长7%，规模以上工业增加值增长6%，实际利用外资增长5%，社会消费品零售总额增长10%，城乡居民人均可支配收入分别增长8%和8.5%，PM2.5平均浓度下降9%，主要污染物减排总量、环境质量控制等全面完成省定目标。

实现上述目标，必须牢牢把握以下五个方面：一是牢牢把握高质量发展根本要求，深刻理解由高速增长向高质量发展阶段转变的科学内涵，将高质量融会贯通进创新、协调、绿色、开放、共享的实践，以高质量发展引领转型升级，以高质量发展打造竞争新优势，以高质量发展赢得未来。二是牢牢把握稳中求进工作总基调，保持稳的节奏，展现进的态势，确保换挡不失速、量增质更优，做到稳中求进、进中求好、增比进位、跨越赶超。三是牢牢把握省委对沧州发展的新定位，围绕打造沿海率先发展创新示范区、建设环渤海地区重要工业城市目标要求，着力发挥沿海临港优势，切实增强产业自信，汇聚先进要素，激发创新活力，推动沿海隆起、城区提升、县域振兴。四是牢牢把握项目建设关键支撑，引导各级牢固树立“抓项目就是抓发展，抓好项目、抓优质项目就是抓高质量发展”的意识，一二三产齐抓、大中小项目齐上，以项目扩总量、提质量，以项目促转型、增后劲。五是牢牢把握决胜全面建成小康社会总目标，抓重点、补短板、强弱项，努力在幼有所育、学有所教、劳有所得、病有所医、老有所养、住有所居、弱有所扶上不断取得新进展。

重点抓好十个方面工作：

（一）抓龙头聚产业，在做强港口经济上实现新突破。沿海有港，是沧州在区域发展中最重要的竞争砝码和胜出优势。我们将突出做好港口建设和渤海新区开发开放两篇文章，加快建成河北沿海经济带高质量发展示范区。

打造国际一流大港。围绕建设现代化综合服务港和国际贸易大港，加强与天津港、河北港口、国家能投三大集团的战略合作，全力抓好总投资341亿元的系列码头群建设，争取30万吨级原油、17.5万立方米LNG等19个码头顺利开工。进一步拓展“直线”、丰富“支线”，完善口岸查验设施，增强通关运营能力，力争全年吞吐量达到3亿吨、集装箱100万标箱，确保黄骅港综合保税区申报成功。加快内陆港建设，大力发展多式联运，放大邯黄、太中银、朔黄铁路三大物流贸易通道集聚效应，全面提升黄骅港面向华北、西北地区的吸引辐射功能。推进337国道、津汕高速黄骅北等“九路三互通”工程，不断完善港口集疏运体系，建设雄安新区和冀中南等广大内陆腹地最便捷的出海口。

聚集高端优质产业。进一步优化园区功能布局，加快构建以先进制造业为主导的现代临港产业体系，重点抓好北交大轨道交通综合研发试验基地、石家庄四药等230个亿元以上产业项目，确保中冶京诚重型装备生产基地、鑫茂华商肉牛养殖加工等60个项目竣工投产，着力打造高端石化、生物医药、汽车零部件和装备制造四大千亿级产业集群。发挥与德国杜伊斯堡、荷兰鹿特丹的“港口战略联盟”作用，推进欧洲中国科技文化商贸中心项目落地实施，建立中国中东欧（沧州）中小企业合作示范区，打造名副其实的“一带一路”北方重要节点、新亚欧大陆桥“桥头堡”。

建设绿色生态新区。坚持生态优先、绿色发展，统筹土地海域资源和环境容量，严格落实港、产、城发展规划和用地用海规划，严把项目质量关、投资强度关，严控污染耗能项目。坚持以城定港、港城融合、产城共兴，进一步优化“一主两副多点”空间布局，抓好157个城市基础设施项目，以及恒大童世界、沧海风景区、欧洲水上小镇、碧桂园生态城等文旅项目，加快打造宜居宜业宜游的现代化滨海新城。

（二）抓对接拓空间，在推进协同发展上实现新突破。京津冀协同发展犹如一座“富矿”，考验河北各地市的智慧和能力。我们将牢牢扭住承接北京非首都功能疏解这个“牛鼻子”，推动产业、交通、生态三个重点领域率先突破，努力在对接京津、服务京津中加快发展自己。

深入开展高层次产业对接。认真落实《京津冀协同发展体制机制改革方案》，健全项目谋划、筛选、洽谈、跟踪等九大机制，开展精准对接行动，做到谋划有方向、合作有目标、洽谈底数清、项目能落地。着眼实现津沧同城化发展，推动落实与天津滨海新区、东丽区、红桥区战略合作协议，力争在园区建设、产业发展等方面形成一批合作成果。抢抓国家审批雄安新区规划框架的契机，进一步加强与雄安新区发展规划的无缝衔接，谋划建设雄安新区科技成果转化和制造业协作基地。着力打造沧州现代产业基地、渤海新区新型工业化基地、任丘石化产业基地三大对接平台，扎实推进京南国家科技成果转移示范区、津冀（沧州）产业转移升级示范区和京津冀天然气协同示范区建设，加快项目落地步伐，扩大规模效益，发挥示范带动效应。

积极承接非首都功能疏解。以京津高校、科研单位为重点，加强教育、医疗、科技、文化、金融等领域合作。灵活采取设立分校、联合办学、共享师资、定向培养等方式，大力吸引京津优质教育资源向我市转移。充分利用合作办医、远程诊疗等手段，积极争取京津知名医疗机构来沧建设分院、开设教学基地。加快推进明珠服饰产业小镇、河北工大科技园等一批在建项目，力争更多非首都功能疏解项目落地建设。

加快推进交通基础设施对接。积极融入全省大交通三年计划，加快津沧、雄沧港、保沧城际铁路和曲港、邯港高速公路等重大项目前期工作，力争石衡沧港城际铁路年内开工，推动沧州与京津和省会的互联互通，建设“轨道上的京津冀”重要交通枢纽。

（三）抓投资上项目，在筑牢发展根基上实现新突破。项目建设是经济工作的总抓手。我们将把2018年作为“项目建设攻坚年”，加压力、增动力，推动形成谋划一批、跑办一批、开工一批、投产一批的良性循环。

抓战略支撑。着眼发挥重大项目“聚集”和“裂变”效应，紧紧抓住45个战略支撑项目，明确时间节点，强化跟踪调度，确保顺利推进。突出抓好华北石化千万吨炼油、神华多式联运智慧物流、北京电力总厂设备搬迁等34个投资超50亿元、过100亿元项目，千方百计抓落地、促开工、保投产。

抓省市重点。进一步充实市、县两级项目库，储备亿元以上项目1000个，力争更多项目进入国家和省建设计划。加大推进力度，确保午易家居物流港、中国陶瓷东星家居建材城等200个亿元以上重点项目开工建设，鸿宇通讯器材、瑞恒机械核电及航天配件等100个项目竣工投产，全年完成产业投资2000亿元以上。

抓督导考核。继续实行重点项目领导分包和观摩考评机制，坚持每月对县（市、区）前期和新建项目进行实地巡查督导，进一步调动全市争项目、跑项目、上项目的积极性和主动性。依托省重点项目三级协调系统，及时解决项目推进中的困难和问题。

（四）抓调整优结构，在促进动能转换上实现新突破。产业转型是事关沧州前途命运的一场硬仗。我们将坚持质量第一、效益优先，坚定不移推动产业向中高端迈进，构建具有沧州特色的现代产业体系。

以优化存量推动转型升级。实施“六个一”工程，做大做强管道装备、包装机械、工艺玻璃等“18+7”特色产业集群，打造一批主业突出、结构优化、竞争力强的“产业新地标”。实施“双百”工程，推进总投资945亿元的129个技改项目，强化宏观指导，优化资源配置，扶持优势骨干企业向更高层次跃升。实施质量品牌提升工程，以创建创新驱动质量强市为抓手，组织开展制造业、农产品等质量提升六大专项行动，鼓励企业“增品种、提品质、创品牌”，加强工业设计，弘扬工匠精神，打造“百年老店”，力促沧州企业由“对标”向“创标、制标”转变。实施“个转企、小升规”晋级工程，促进入区进园、集聚发展，全年培育“专精特新”中小企业15家，新增规上工业企业100家。

以优质增量引领转型升级。加快发展清洁能源、信息技术、生命健康、节能环保等战略性新兴产业，安排专项支持资金1亿元，扶持项目100个，重点抓好激光产业园、汽车产业基地、再制造产业基地等园区建设，战略性新兴产业增加值增长10%。大力发展共享经济、数字经济、现代供应链等新业态新模式，积极培育新的增长点。

以服务业拓展促进转型升级。推进实施消费促进、电子商务进农村进社区、商贸物流配送体系建设、实体商业转型升级四大专项行动，依托新华区传化智能公路港、高新区京津服务业转移基地等龙头项目和特色专业园区，大力发展现代物流、电子商务、文化旅游、家政服务等现代服务业，年内新增规上服务业企业90家、增加值增长10%以上。科学编制《沧州市全域旅游发展规划》，启动以吴桥杂技大世界5A级景区创建为引领的高品质景区创建工作，重点抓好沧州武术文化园、河间景和田园综合体等十大旅游项目。精心组织第二届沧州旅游产业发展大会，发掘利用沧州武术、杂技和大运河等特色文化元素，建设沧南旅游示范片区，展示“千年江湖梦、百里运河图”独特魅力，力争全年旅游业总收入增长25%以上。

以军民融合带动转型升级。认真落实《关于推进军民深度融合发展的实施意见》，推进军民科技成果双向转化和共享，深化与军工央企、涉军院校、军队单位的战略合作，重点在航空航天、高技术船舶、新一代电子信息、功能性复合材料等领域，实施一批关键技术研发项目。抓好泊头、高新区等4个省级军民融合创新示范园区建设，年内军民融合企业达到90家。

企业是转型发展的主力军，是社会财富的创造者。我们要更好发挥政策导向作用，打造一流营商环境，培育更多有战略眼光、有社会责任感的企业家，让明星企业、领军企业根深叶茂、四季常青。

（五）抓创新强支撑，在提升竞争实力上实现新突破。创新是经济发展的核心动力。我们将着力发挥科技创新在全面创新中的引领作用，把金融、双创、大智移云作为高质量发展的公共服务基础支撑来打造，积极创建国家创新型城市。

全面夯实创新基础。启动实施科技创新三年行动计划，力争全社会研发投入强度年均增长0.5个百分点。积极推进河北科光宽禁带化合物半导体、中科院沧州纳米研究院等高新技术项目建设，年内新增高新技术企业90家、科技型中小企业1200家。在关键领域组建一批产学研创新联盟，实施国家和省科技项目50项以上、转化科技成果40项以上。加快北京计算机中心黄骅分中心、北京化工大学沧州技术转移中心等创新服务平台建设，新增省级以上工程技术研究中心4家、院士工作站3家、“星创天地”5家。

加快发展大智移云。以“互联网+”行动计划和“大智移云”引领计划为抓手，加快建设高速、移动、安全、泛在的新一代信息基础设施。深化与华为、阿里巴巴、九次方、微软、浪潮等信息产业巨头合作，支持大数据产业公司发展壮大，抓好华为大数据云计算中心、城市大数据中心和政务服务平台建设，促进信息集成、数据共享，把沧州推向“云”时代。

着力加强金融创新。深入落实加强金融产品创新、支持实体经济发展的政策措施，大力发展科技金融、普惠金融、绿色金融，力争全市金融机构存贷比达到63%。加大上市企业培育力度，年内新增挂牌上市企业15家。壮大政府引导基金规模，用好用活金融控股公司平台，进一步拓展资产管理、信托和投行类金融服务空间。加强信用体系建设，创优金融生态环境，构建和谐政银企关系。

持续释放“双创”活力。坚持创新创业结合、线上线下结合、孵化转化结合，推广众创、众包、众扶、众筹商业模式，鼓励大型企业、高等院校创办各类众创空间，突出抓好北京路“双创一条街”建设，新增省级科技孵化器2家、众创空间7家以上。办好沧州市第三届青年创新创业大赛，支持能人创业、草根创业、返乡创业，点燃沧州激情、汇聚创新力量。

人才是创新的核心要素。一个科技日新月异、发展突飞猛进的时代，离不开人才的有力支撑。我们要加快启动“人才兴沧”工程，实施精英引进计划，努力用好政策吸引人才、用好机制激励人才、用好环境留住人才，把沧州打造成创新蓝海、创业沃土、创客乐园。

（六）抓城镇重统筹，在扩容提质升级上实现新突破。推进新型城镇化，以全新的形象融入京津冀世界级城市群，是沧州现代化进程中的一件大事。我们将坚持世界眼光、国际视野、战略思维，对标国际国内一流城市，推动区划调整，加大建设投入，丰富城市内涵，不断提升城镇发展的质量和水平。

提升城区承载能力。以创建生态园林城市、智慧城市、海绵城市、文明城市为抓手，着眼东西部均衡发展，重点实施园林绿化、道路畅通等总投资576亿元的12大类133个城建项目，打造运动休闲、整洁有序、景观优美、生态宜居的现代化中心城区。按照“中国大运河文化重要承载地、河北省城市生态休闲走廊示范区、沧州市城市重要标志”的目标定位，加快推进大运河生态公园一期项目建设，确保年内见成效、出亮点。完成名人植物园二期、滨河公园二期等19个公园绿地项目，建设“大化·1973”文化主题园，新增绿地17.2万平方米。投资3亿元，分三年对中心城区400个老旧小区道路、绿化、排水及墙体设施实施提升改造，年内先行改造小区200个。新建续建南北绕城、贵州大道等44条城市道路，打通“断头路”、推进“大外环”，构建完整闭合的城市交通网络。规范化改造菜市场11家，有效解决市场“脏、乱、差”和市民“买菜难”问题。

培育做大城市经济。依托“三区两园”，大力发展高新技术产业和高端服务业，重点抓好华北（沧州）高端智能装备产业园、核燃料产业园等项目建设。支持沧州开发区做大汽车产业规模，向千亿级园区迈进。积极发展总部经济、楼宇经济，打造一批税收超千万元的商务楼、写字楼。培育壮大颐和广场、荣盛广场、千童广场等重点商圈规模，推进高端城市商业综合体建设，支持大型商贸企业和特色街区开展夜购、夜娱等消费服务，进一步活跃城市夜经济。

推进县城扩容提质。深入开展县城建设攻坚行动，按照“小县大县城”发展思路，进一步拉大整体框架、完善县城功能，推动产城教融合发展，力争任丘、青县、献县通过国家园林城市（县城）初检。按照“即拆、即清、即整、即建、即美”原则，大力开展清理违法占地和“一区三边”违法建设专项整治行动。加快沧州主城区至黄骅市、任丘市快速路前期工作，织密县际交通网络。积极推进乡改镇、镇改街、村改居，年内转移农村人口16.2万人，力争全市常住人口城镇化率提高2个百分点。进一步延伸产业链条，壮大特色产业规模，促进县域经济增比进位，主营业务收入超百亿元的产业集群达到12个，一般公共预算收入超10亿元的县（市）达到8个。

优化城市综合管理。全面推进环卫市场化运营和城市执法体制改革，建立精细化、标准化、智能化城市管理系统，推动行政处罚权相对集中、管理权向辖区延伸，确保城市管理无死角、全覆盖。大力开展城市环境综合整治行动，对246条小街小巷进行集中改造，坚决取缔店外占道经营、垃圾乱扔乱倒等不文明行为，打造美丽街道、靓丽街区，市区清扫保洁机械化率达到90%以上。加快实施“智慧泊车”项目，中心城区新建智能化公共停车场6个、新增泊位2065个。推广应用“全民城管APP”，让每位市民成为城市管理的参与者和监督员。

（七）抓农业夯基础，在推进乡村振兴上实现新突破。农村大地是充满希望的热土，农村工作是大有作为的舞台。我们将大力实施乡村振兴战略，努力使农民成为体面的职业、农业成为有奔头的产业、农村成为令人向往的美丽家园。

加快推动农业全面升级。启动农业供给侧结构性改革三年行动计划，加快发展质量农业、绿色农业、都市型农业。在确保粮食安全的前提下，实施“南棉北菜东草西特”工程，着力提升绿色优质农产品供给能力。全面推行标准化生产，抓好8个农产品质量安全县创建。推进“互联网+现代农业”，构建全产业链技术支撑体系，促进一二三产融合发展。积极培育专业大户、家庭农场、农民专业合作社等新型主体。加快现代农业园区建设，培育2个以上省级区域公用品牌，抓好盐山新发地、黄骅正邦等50个超亿元农业产业化项目，产业化经营率达到66.5%以上。

加快推动农村全面进步。建立健全实施乡村振兴战略财政投入保障制度，加大金融保险服务“三农”力度，拓宽资金筹集渠道。深入落实承包地“三权”分置制度，加快农村产权流转交易市场体系建设，实现县级交易中心全覆盖，全市土地流转率达到45%以上，60%的行政村完成清产核资任务。开展农村人居环境整治三年行动，大力推进产业园区、生态园区、农村新型社区“三区同建”，建设示范区50个；加快“四好农村路”建设，新改建农村公路500公里；启动实施农村生产危桥改造和坑塘整治提升三年计划，改造维修危桥120座、整治提升坑塘3000个；推进“厕所革命”，农村卫生厕所普及率达到75%以上。创新乡村治理体系，深化村民自治实践，建设平安乡村，确保乡村社会充满活力、和谐有序。

加快推动农民全面发展。实施农业农村“领头雁”计划和一村一名大学生计划，加强农业技术人才引进，鼓励高校毕业生、退伍军人、科技人员到农村施展才华，培养造就一支懂农业、爱农村、爱农民的“三农”工作队伍，带领群众创业致富。加强新型职业农民技能培训，引导支持农民就业创业，挖掘经营性收入增长潜力，稳住工资性收入增长势头，释放财产性收入增长红利，拓展转移性收入增长空间，促进农民收入稳步增长、过上富裕生活。

（八）抓改革促开放，在增强内生动力上实现新突破。改革引领时代，开放赢得舞台。我们将坚决把改革进行到底，把开放的大门敞得更开。

深入推进供给侧结构性改革。大力破除无效供给，运用市场、标准、法治等手段，严控过剩行业新上产能。有效控制国有企业债务，支持企业去杠杆、债转股。坚持住房的居住属性，合理有序供应土地，积极构建高端有市场、中端有供应、低端有保障的供给体系，促进房地产市场平稳健康发展。开展降费减负专项行动，大力降低制度性交易成本、用能和物流成本。加快补齐公共服务、基础设施、民生保障等领域短板。

统筹推进重点领域改革。深化国有企业改革，加大国有企业混合所有制改革力度，重点做好70家市属危困企业改革退出工作，全面完成国有企业“三供一业”分离移交工作，市级经营性国有资产实现集中统一监管。深化投融资体制改革，更加注重投资强度和质量，不断提高投资回报率、资源利用率、土地使用率、劳动生产率。放宽民间资本市场准入领域，完善政府和社会资本投资模式，激发民间投资活力。深化绩效预算管理和财政事权与支出责任划分改革，创新财政支持产业发展方式，增强财政资金的引导和放大作用。深化科技投入、成果转化、收益分配改革，加强知识产权创造、保护、运用。统筹推进教育、文化、医药卫生等领域改革，充分释放改革红利。

加快转变政府职能。大力推进简政放权，推动“多证合一”扩容升级，实施市场准入负面清单制度，着力提升“双随机、一公开”监管规范化、标准化水平。加快市县行政审批局和“四级网上审批与服务”建设，建成全市统一的一体化在线服务平台，力争全流程网上办理和“最多跑一次”办理的事项比例不低于40%，最大程度便利企业、惠及群众。

拓展开放深度广度。把招商引资作为重要任务，力争全年洽谈引进150个亿元以上项目。注重产业招商，以县（市、区）、开发区为主体，编制产业招商规划，实行“一个产业、一名领导、一个团队、一张招商图”机制，引进一批建链、强链、补链项目。注重平台招商，利用武术、杂技等传统节庆活动资源，发挥河间、肃宁、东光、中捷等展会品牌优势，谋划搭建主题突出、有较强影响力的对外招商推介平台，瞄准国内外500强和行业领军企业，精准开展大规模招商引资。注重以商招商、社会化招商，实现政府与企业、与专业机构的协调联动，吸引更多高端优质项目落户。进一步深化开发区体制机制改革，壮大开发区规模层次，省级以上开发区主要指标增速达到15%以上。围绕对接“一带一路”，做强做优特色出口基地，力争新增进出口超千万美元企业5家以上，进出口贸易总额突破260亿元。

（九）抓重点补短板，在打好三大攻坚战上实现新突破。打好防范化解重大风险、精准脱贫、污染防治三大攻坚战，事关实现全面建成小康社会目标。我们将坚持问题导向、底线思维，瞄准重点难点，精准发力、务求实效。

打好防范化解重大风险攻坚战。有效防控金融风险，严厉打击非法集资、违规融资担保行为，坚决守住不发生系统性、区域性金融风险底线。有效防控地方债务风险，合理控制政府性债务规模。有效防控安全生产风险，启动实施“卡单函令”制度，深入开展危险化学品、油气管网、建筑施工等高危行业和领域专项整治，加强道路交通安全和消防安全管理，坚决遏制各类安全事故发生。有效防控食品药品安全风险，扎实推进“双安双创”工作，保障人民群众饮食用药安全。有效防控公共安全风险，完善社会矛盾预防和排查化解综合机制，用法治思维和法治方式化解社会矛盾、解决群众利益诉求。深入开展扫黑除恶专项斗争，依法严厉打击各类违法犯罪行为，为人民群众创造和谐稳定、安全有序的社会环境。

打好精准脱贫攻坚战。把脱贫攻坚作为头等大事和第一民生工程，确保1.88万贫困人口稳定脱贫、盐山脱贫摘帽。认真落实《关于加大非贫困县（市）脱贫攻坚推进力度的意见》，加快推进政策、项目、资金向非贫困县（市）贫困人口倾斜。实施产业扶贫攻坚行动和“百企帮百村”活动，统筹用好各项扶持政策，加快发展“牧菜菌”、光伏发电、农村电商等扶贫产业，力争国家级贫困县光伏电站全覆盖。深入开展扶贫领域腐败和作风问题专项整治，强化资金监管，严格追责问责。严把贫困人口退出关口，决不搞虚假脱贫、数字脱贫，确保脱真贫、真脱贫。

打好污染防治攻坚战。经历雾霾之痛，更觉蓝天珍贵。坚持把冬季治霾作为重中之重，推动落实冬季清洁取暖三年规划，年内实施气（电）代煤8.58万户，千方百计保障气源和电力供应，确保群众温暖过冬。强化控煤、限车、治企、抑尘、禁烧等五个方面10条措施，确保上级交办环境问题按时整改到位。充分发挥大气污染热点网格和重点企业在线监控作用，严防严控、严管严治，确保老问题不反弹、新问题不出现，真正提升“蓝天幸福感”。严格落实“水10条”与“河长制”，加强重污染河流、城市黑臭水体综合整治，持续修复近岸海域和湿地生态，重现小河流水、湿地润城、海天一色的自然天韵。全面实施“土10条”，加快推进土壤环境污染状况调查评估，对城市污染场地、受污染农用地进行专项治理。大力开展国土绿化三年行动，瞄准“三年攻坚、造林百万”目标，突出抓好大运河沿线、中心城区高速围合区等五大绿化工程，年内新增造林面积40万亩以上。深入开展“2018利剑斩污”专项行动，以“零容忍”的态度坚决查处环境违法行为。启动实施《环境空气质量通报排名和奖惩问责办法》，始终保持督办问责的高压态势。

绿水青山就是金山银山。我们一定要像对待生命一样对待生态环境，把蓝天碧水、秀美风光永远留在沧州大地，留给子孙后代！

（十）抓民生增福祉，在创造美好生活上实现新突破。为政之道，民生为本。我们将坚持人民至上、共建共享，努力使人民群众的获得感、幸福感、安全感更加充实、更有保障。今年重点实施学前和中小学教育提升、城区便民菜市场建设、食品药品安全、公立医院改革、社区和居家养老、城乡特困人员救助、农村危房改造、棚户区改造、老旧小区改造、就业和再就业、公园绿地建设、运动休闲城市建设、交通秩序整治、城乡厕所改造、环卫市场化管理、冬季清洁取暖、公共文化服务、政务便民服务等18项民生工程，逐个制定专项实施方案、保障措施，确保每项工程高标准、高质量完成。

优先发展教育事业。启动第三期学前教育行动计划，吸引社会力量举办托幼机构，新改扩建公办幼儿园80所。加快市县城区教育资源增量扩容，推进城乡义务教育均衡发展，大力改善薄弱学校办学条件，新改扩建中小学169所。实施高中阶段教育普及攻坚计划，着力提升教育教学质量。深化职业教育产教融合、校企合作，扎实推进“双扩大”工程，确保市属高职院校生均拨款标准不低于12000元。支持高校优化学科布局和专业设置，建设省内一流学科。加强师德师风建设，培养高素质教师队伍，倡导全社会尊师重教。完善困难学生资助体系，着力解决中小学生课外负担重、“择校热”“大班额”等突出问题，办好人民满意的教育。

提高就业质量水平。改革就业培训机制，加强全方位就业服务，扎实做好企业分流职工、高校毕业生、就业困难人员等重点群体安置工作，年内新增城镇就业6万人，城镇登记失业率控制在4.5%以下。支持农村劳动力转移就业和农民工返乡创业，严厉打击拖欠农民工工资行为。

加强社保体系建设。深入实施全民参保计划，推进机关事业单位养老保险制度改革，新增城乡养老保险参保人数1.6万人，企业和机关事业单位退休人员基本养老金增长5%。完善最低生活保障和社会救助制度，健全农村留守儿童和妇女、老人关爱服务体系，落实好特困人员、残疾人、优抚对象等群体的兜底保障，城乡居民低保标准分别提高到7440元和4200元。

推进健康沧州建设。坚持“三医联动”，深化医药卫生体制改革，扩大公立医院改革成效，完善分级诊疗差别化医保支付制度，落实药品采购“两票制”，严格控制医疗费用不合理增长。推进医联体建设和分级诊疗，扩大家庭医生服务签约范围。大力发展中医药事业，启动沧州市中医院、中西医结合医院骨伤康复院区等项目建设。继续做好计划生育工作。鼓励社会资本兴办养老机构，加快推进医养结合型养老示范基地建设，城市社区居家养老服务设施覆盖率达到85%，新建养老院、互助幸福院123所。

保障基本住房需求。实施新的三年棚户区改造计划，年内新开工8000套。加大公租房保障力度，对低保、低收入住房困难家庭加快实现应保尽保。继续开展农村危房改造，针对建档立卡贫困户、低保户、分散供养特困户、贫困残疾人户“四类农户”完成改造任务1674户。

繁荣文化体育事业。大力推进运动休闲城市建设，围绕构建中心城区“8分钟健身圈”，新建气膜网羽馆、滑冰馆、沧州体育运动学校综合训练馆、沧州少儿体校综合训练馆等四座市级体育馆，新建改造体育设施93处；全市新增体育场地面积71万平方米，为500个行政村配备体育健身设施。精心组织好河北省第十届少数民族传统体育运动会、中国沧州国际武术节等系列体育赛事。深入推进国家公共文化服务体系示范区创建工作，市县乡村普遍达到“五个有”标准。加快市工人文化宫建设，实施“文化干细胞”培育工程和文体惠民工程，年内新改扩建图书馆9个、文化馆8个。

弘扬拥军优属、拥政爱民的优良传统，加强国防教育和国防后备力量建设，巩固军政军民团结的好局面。充分发挥工会、共青团、妇联等人民团体桥梁纽带作用。继续做好民族宗教、外事侨务、人民防空、气象地震、史志档案等工作。

各位代表，人民对美好生活的期待，就是我们的奋斗目标，这是庄严承诺，也是施政箴言。我们一定铭记总书记教导，恪尽职守，勤勉工作，努力推动沧州实现更高质量、更有效率、更加公平、更可持续的发展！

三、全面加强政府自身建设

新时代赋予新使命，新征程召唤新作为。我们将紧跟时代步伐，牢记为民宗旨，扎实开展“双创双服”活动，全力打造勤政务实、廉洁高效、人民满意政府。

（一）始终秉持炽热情怀，以许党许国的使命担当展现时代新作为。深入贯彻新时代党的建设总要求，树牢“四个意识”，提高政治站位，在思想上行动上同以习近平同志为核心的党中央保持高度一致。坚持党对一切工作的领导，做到党的工作部署到哪里，政府工作就推进到哪里，力量就汇聚到哪里。坚持知行合一、勇毅笃行，以为党分忧、为国尽责、为民奉献的情怀和担当，把聪明才智用在工作上，把心血精力用在发展上，做守土尽责的坚强“卫士”、服务发展服务人民的模范“标兵”。

（二）始终保持昂扬斗志，以敢为人先的气魄胆略拓展发展新空间。从市政府做起，坚决不搞虚报浮夸的表面文章，坚决不搞华而不实的花拳绣腿，不驰于空想、不骛于虚声，迎着困难上，奔着问题去，靠一个个矛盾的解决、一个个难题的破解，为沧州的发展打开一片新天地。坚持事争一流、高位对标，敢于瞄准强者争先进位，善于发现问题突破自己，努力推进各项重点工作争当全省排头兵。树立打基础、利长远工作导向，以功成不必在我的境界，稳扎稳打、善做善成，为沧州未来发展夯基垒台。

（三）始终坚持实干实政，以勤勉敬业的过硬作风抓出工作新成效。建立日常学习和定期调研制度，通过真学细研深悟，解放思想、转变观念、提高本领。按照“一分部署、九分落实”的要求，拧牢责任螺栓、上紧督查发条、用好问责利剑，坚决纠正表态多调门高、行动少落实差等突出问题。推行政府工作台账管理，对重大工程和重要事项超前策划、科学安排，在推进过程中多打主动仗、多打提前量，防止前松后紧、被动突击。认真执行“日清日结”工作制度，决不允许文件循环运转、问题久拖不决，做到事事有着落、件件有结果。建立健全层层负责、压茬推进的督导检查机制，瞄准“牵一发动全身”的重点工作，督任务、督进度、督成效，查责任、查作风、查状态，一锤接着一锤敲，一茬接着一茬干，确保日有所进、月有所成，每年都有大变化。

（四）始终守持纪律规矩，以风清气正的政治生态树立政府新形象。锲而不舍落实中央“八项规定”，瞄准重点领域廉政风险点，加大行政监察和审计监督力度，以更高标准抓好政府系统反腐倡廉工作。大力推进作风建设，深入开展“十查十改”，持之以恒克服形式主义、官僚主义，久久为功袪除享乐主义、奢靡之风。强化依法行政，自觉接受人大的法律监督和政协的民主监督，虚心接受社会监督和舆论监督，严格落实民主集中制和“三重一大”议事规则，依法依规依程序行使职权、履行职责，让权力在阳光下运行。

各位代表，习近平总书记在党的十九大报告中指出，“历史车轮滚滚向前，时代潮流浩浩荡荡。历史只会眷顾坚定者、奋进者、搏击者，而不会等待犹豫者、懈怠者、畏难者”。我们深知，全体政府工作人员，肩负着时代的重任，承载着人民的期盼。我们一定以永不懈怠的精神状态和一往无前的奋斗姿态，攻坚克难、创优争先，在中共沧州市委的坚强领导下，奋力走好新时代的长征路，书写中华民族伟大复兴中国梦的沧州篇章。让我们更加紧密地团结在以习近平同志为核心的党中央周围，不忘初心、牢记使命，埋头苦干、锐意进取，为开创新时代建设创新驱动经济强市、生态宜居美丽沧州新局面而努力奋斗！
